# Cancer incidence attributable to tobacco smoking in GCC countries in 2018

**DOI:** 10.18332/tid/118722

**Published:** 2020-03-19

**Authors:** Abdulmohsen H. Al-Zalabani

**Affiliations:** 1 Department of Family and Community Medicine, College of Medicine, Taibah University, Al-Madinah, Saudi Arabia

**Keywords:** cancer, lifestyle, tobacco smoking, primary prevention, population attributable fraction

## Abstract

**INTRODUCTION:**

The Gulf Cooperation Council (GCC) member countries include Bahrain, Kuwait, Oman, Qatar, Saudi Arabia, and United Arab Emirates. The current study aims to provide an estimate of the population fractions of cancer cases attributable to tobacco smoking in the GCC countries.

**METHODS:**

Population attributable fraction (PAF) was calculated for cancers that were listed by the International Agency for Research on Cancer (IARC) to have sufficient evidence of causal association. The estimated number of incident cancer cases in GCC countries were retrieved from the IARC GLOBOCAN database. The prevalence estimates of current tobacco smoking among persons aged ≥15 years were obtained from the World Health Organization report on prevalence of tobacco smoking. Relative risk estimates for various cancers were obtained from published meta-analyses. Summary PAFs and cancer cases attributable to tobacco smoking are reported by country, sex, and cancer type.

**RESULTS:**

Tobacco smoking was responsible for 2536 (16.3%) of cancer cases in GCC countries in 2018. It accounted for 22.8% (n=2396) and 2.8% (n=140) of cancer cases among males and females, respectively. Among males, the highest number of cancer incident cases attributable to smoking was lung cancer (807) followed by urinary bladder (328), and colorectal cancer (305). Among females, the highest number of cancer cases attributable to smoking was lung cancer (62) followed by lip and oral cavity (13), and cervical cancer (13).

**CONCLUSIONS:**

Tobacco smoking accounted for a large portion of cancer cases attributable to preventable risk factors in GCC countries. Preventive efforts focusing on reducing tobacco smoking should be a high priority in GCC countries.

## INTRODUCTION

Tobacco smoking is a major risk factor for many types of cancer. The International Agency for Research on Cancer (IARC) stated that there is sufficient evidence that tobacco smoking causes the following cancers: lung, oral cavity, nasal cavity and paranasal sinuses, nasopharynx, oropharynx, hypopharynx, larynx, oesophagus, stomach, pancreas, liver, colorectum, kidney, ureter, urinary bladder, cervix, ovary (mucinous type), and myeloid leukaemia^1^. Tobacco smoking contributes a large share of the cancer burden. This is because tobacco smoking has a substantial prevalence, is associated with several types of cancer, and has high relative risk for cancer^1^.

The Gulf Cooperation Council (GCC) member countries include Bahrain, Kuwait, Oman, Qatar, Saudi Arabia, and United Arab Emirates. The GCC population is estimated to be about 56 million, ranging from 1.5 million in Bahrain to 33.4 million in Saudi Arabia^2^. GCC countries experienced a rapid modernization and urbanization leading to vast changes in the profile of the disease burden and rapid increase in the risk factors of non-communicable diseases, with obesity and physical inactivity reaching alarming levels^3^. In the GCC countries, 38404 new cancer cases and 17211 cancer deaths are estimated to have occurred in 2018^4^. Liver, lung, colorectal and breast cancers were among the top 25 causes of death in GCC countries in 2010^5^. Moreover, the incidence of cancer has increased in recent decades in the GCC countries^6^. This increase could be partially explained by the aging population, improved diagnosis, and improved reporting of cancer cases. However, the main driving force is more likely to be the changes in the prevalence of risk factors among the GCC populations, including tobacco smoking, which showed a steady increase since 2000, except in Kuwait^3,7^.

Quantifying the contribution of various risk factors in the burden of cancer is vital for the success of prevention efforts. Thus, the aim of the current study was to provide an estimate of the population fractions of cancer cases attributable to tobacco smoking in the GCC countries.

## METHODS

Measures of population impact, like population attributable fraction (PAF), are commonly used to quantify the proportion of incidence cases of cancer caused by a certain risk factor^8–10^. PAF is specific to each population and assumes causal relationship between the risk factors and the disease. To calculate PAF, certain data are required including prevalence of risk factors in the population, measure of strength of association for each risk factor, and estimates of cancer cases in the same population.

### Selection of cancers

Cancers selected to be included in the current analysis are cancers that were listed by IARC to have sufficient evidence of causal association with exposure to tobacco smoking. These cancers are: oral cavity, nasopharynx, oropharynx, hypopharynx, oesophagus, stomach, colorectum, liver, pancreas, larynx, lung, uterine cervix, urinary bladder, kidney, and myeloid leukaemia^1^.

Mucinous type of ovarian cancer and ureter cancer were also listed in the IARC list of tobacco-related cancers, but their data were not available in the GLOBOCAN database. Ovary cancer is reported in the GLOBOCAN database as a one category with no details of subtypes and thus the number of cases could not be used in the current analysis because mucinous type constitutes only 10% of all ovarian cancer^11^. Similarly, cancer of the ureter was only reported as part of all cancers and thus it was not possible to include it in the current analysis.

### Cancer incidence

The estimated number of incident cancer cases in GCC countries were retrieved from the IARC GLOBOCAN database^4^ using its interactive interface ‘Cancer Today’. The GLOBOCAN database provides estimates of incidence, prevalence and mortality of cancer by site and sex for all countries in 2018. The data are based on reports from the national cancer registry of each country and projected to the 2018 population of the country^4^.

The number of cases is reported separately for each type of cancer, except myeloid leukaemia which was reported within a broader group of leukaemia that includes other types of leukaemia. To estimate the proportion of myeloid leukaemia in the overall leukaemia category, data from the Cancer Incidence in Five Continents (CI5), Volume XI, were used^12^. CI5 data were available for four countries (Bahrain, Kuwait, Qatar, and Saudi Arabia) and the proportion was calculated for each country using these data. The average proportion of the four countries was used as an estimate for the proportion of myeloid leukaemia for the other two countries (Oman, and United Arab Emirates).

### Prevalence of exposure

The prevalence estimates of current tobacco smoking among persons aged ≥15 years were obtained from the ‘WHO global report on trends in prevalence of tobacco smoking 2000–2025^7^. The report used data from national surveys, conducted in each country in the period 1990–2015, to run statistical models to estimate the prevalence rates and the credible interval for the estimate. It is well known that cancer is caused by past exposure to various risk factors rather than immediate exposure. The period between exposure to a risk factor and cancer diagnosis is known as the latency period. To have a balance between reasonable latency period and available good quality exposure data^10^, estimates of prevalence of tobacco smoking in the year 2005 in GCC countries (13-year lag) were used in the current calculations.

### Relative risk estimates

Relative risk (RR) estimates for various cancers were obtained from published literature. Most of the estimates were obtained from a meta-analysis published by Gandini et al.^13^, which included 216 original studies. Separate estimates for males and females were used in the calculations for cancer sites for which were provided estimates. If the overall estimates only were provided, the overall estimates were used for both males and females. Colorectal cancer was not included in their main analysis. Thus, the RR estimate for colorectal cancer was retrieved from another large meta-analysis^14^. Moreover, the RR estimate for myeloid leukaemia was retrieved from a more comprehensive meta-analysis^15^.

### Statistical analyses

Estimates of prevalence of current tobacco smoking in each GCC country and RR estimates identified in previous meta-analyses were used to calculate the PAF for each type of cancer. The following formula was used:

PAF=[(RR−1)−P]/[1+(RR−1)−P]

where RR is the relative risk for a cancer due to current smoking, and P is the proportion of the population that were current smokers at the time of exposure estimate.

Cancer cases attributable to tobacco smoking were calculated by multiplying the number of observed cancer cases in each country with the corresponding PAF of that country.

## RESULTS

Table 1 presents the prevalence estimates of current smoking among adults in the GCC countries. The highest prevalence among males was observed in Kuwait (40.5%) followed by the United Arab Emirates (34.9%), and Bahrain (33.2%). The highest prevalence among females was observed in Bahrain (6.8%) followed by Kuwait (4.4%). Oman had the lowest prevalence of current smoking among both males (14.2%) and females (0.6%). The estimates of RR of each type of cancer attributable to current smoking are shown in Table 2. They show that cancers of lung, larynx and pharynx have the highest RR for current tobacco smoking.

**Table 1:** Tobacco smoking prevalence in GCC member countries, 2005

*Country*	*Population (million)*	Prevalence* (%)	

		*Male*	*Female*
Bahrain	1.5	33.2	6.8
Kuwait	4.4	40.5	4.4
Oman	4.6	14.2	0.6
Qatar	2.8	25.0	1.4
Saudi Arabia	33.4	23.6	3.1
United Arab Emirates	9.3	34.9	1.6

* Source: ‘WHO global report on trends in prevalence of tobacco smoking 2000–2025^7^.

**Table 2:** Relative risks of cancers for current tobacco smoking

*Cancer site*	*RR (95% CI)*		

	*Men*	*Women*	*All*
Lip and oral cavity	-	-	3.43 (2.37–4.94)
Nasopharynx	-	-	1.95 (1.31–2.91)
Pharynx (Hypo+Oro)	-	-	6.76 (2.86–16.0)
Oesophagus	2.52 (1.81–3.52)	2.28 (1.51–3.44)	2.50 (2.00–3.13)
Stomach	1.74 (1.46–2.07)	1.45 (1.20–1.75)	1.64 (1.37–1.95)
Colorectum^a^	1.38 (1.22–1.56)	1.06 (0.95–1.19)	1.20 (1.10–1.30)
Liver	1.85 (1.21–2.83)	1.49 (1.12–1.98)	1.56 (1.29–1.87)
Pancreas	1.63 (1.32–2.03)	1.73 (1.31–2.30)	1.70 (1.51–1.91)
Larynx	-	-	6.98 (3.14–15.5)
Lung	9.87 (6.85–14.24)	7.58 (5.36–10.73)	8.96 (6.73–12.1)
Urinary bladder	2.80 (2.01–3.92)	2.73 (1.82–4.10)	2.77 (2.17–3.54)
Kidney	1.59 (1.32–1.91)	1.35 (1.05–1.73)	1.52 (1.33–1.74)
Myeloid leukaemia^b^	-	-	1.09 (0.70–1.70)

Source of RR data, Gandini et al.^13^. Other sources: a Tsoi et al.^14^ and b Fircanis et al.^15^.

PAF estimates for current tobacco smoking for each country by sex are presented in Table 3. As shown, tobacco smoking accounted for 55.7% to 78.2% of lung cancers among males in the GCC countries. Oman has the lowest PAF for all cancer types among males and females due to the low prevalence of current smoking. For all types of cancer, Kuwait has the highest PAF among men, while Bahrain has the highest PAF among women.

**Table 3:** Proportion (%) of cancer cases attributable to current smoking by sex and cancer sites, GCC countries, 2018

*Cancer site*	*Bahrain*	*Kuwait*	*Oman*	*Qatar*	*Saudi Arabia*	*UAE*
**Men**						
Lip and oral cavity	44.7	49.6	25.7	37.8	36.4	45.9
Nasopharynx	24.0	27.8	11.9	19.2	18.3	24.9
Pharynx (Hypo+Oro)	65.7	70.0	45.0	59.0	57.6	66.8
Oesophagus	33.5	38.1	17.8	27.5	26.4	34.7
Stomach	19.7	23.1	9.5	15.6	14.9	20.5
Colorectum	11.2	13.3	5.1	8.7	8.2	11.7
Liver	22.0	25.6	10.8	17.5	16.7	22.9
Pancreas	17.3	20.3	8.2	13.6	12.9	18.0
Larynx	66.5	70.8	45.9	59.9	58.5	67.6
Lung	74.7	78.2	55.7	68.9	67.7	75.6
Urinary bladder	37.4	42.2	20.4	31.0	29.8	38.6
Kidney	16.4	19.3	7.7	12.9	12.2	17.1
Myeloid leukaemia	11.7	13.9	5.4	9.1	8.6	12.2
**Women**						
Lip and oral cavity	14.2	9.7	1.4	3.3	7.0	3.7
Nasopharynx	6.1	4.0	0.6	1.3	2.9	1.5
Pharynx (Hypo+Oro)	28.1	20.2	3.3	7.5	15.2	8.4
Oesophagus	8.0	5.3	0.8	1.8	3.8	2.0
Stomach	3.0	1.9	0.3	0.6	1.4	0.7
Colorectum	0.4	0.3	0.0	0.1	0.2	0.1
Liver	3.2	2.1	0.3	0.7	1.5	0.8
Pancreas	4.7	3.1	0.4	1.0	2.2	1.2
Larynx	28.9	20.8	3.5	7.7	15.6	8.7
Lung	30.9	22.5	3.8	8.4	16.9	9.5
Uterine cervix	5.3	3.5	0.5	1.1	2.5	1.3
Urinary bladder	10.5	7.1	1.0	2.4	5.1	2.7
Kidney	2.3	1.5	0.2	0.5	1.1	0.6
Myeloid leukaemia	2.6	1.7	0.2	0.6	1.2	0.6

Finally, cancer incidence in the GCC countries attributable to smoking is given in Table 4. Tobacco smoking was responsible for 2536 (16.3%) of cancer cases in the GCC countries in 2018 (2396 male cases and 140 female cases). Among males, the highest number of cancer cases attributable to smoking was lung cancer (807) followed by urinary bladder (328), and colorectal cancer (305). Among females, the highest number of cancer cases attributable to smoking was lung cancer (62) followed by lip and oral cavity (13), and cervical cancer (13).

**Table 4:** Cancer observed cases and cases attributable to tobacco smoking in GCC countries, 2018

*Cancer site*	*Bahrain*						

	*Bahrain*	*Kuwait*	*Oman*	*Qatar*	*Saudi Arabia*	*UAE*	*Total*
**Men**							
Lip and oral cavity	5 (12)	19 (38)	13 (50)	8 (22)	79 (216)	6 (13)	130 (351)
Nasopharynx	1 (4)	6 (21)	2 (19)	2 (8)	59 (321)	1 (4)	70 (377)
Pharynx (Hypo+Oro)	1 (1)	2 (3)	9 (20)	1 (2)	17 (29)	5 (8)	35 (63)
Oesophagus	2 (5)	8 (22)	3 (17)	2 (8)	43 (164)	9 (27)	68 (243)
Stomach	3 (16)	10 (43)	15 (157)	5 (31)	53 (358)	15 (73)	101 (678)
Colorectum	8 (72)	26 (195)	13 (261)	9 (100)	198 (2404)	51 (436)	305 (3468)
Liver	4 (20)	23 (88)	9 (88)	6 (37)	112 (668)	14 (61)	168 (962)
Pancreas	3 (18)	9 (46)	5 (55)	2 (15)	50 (386)	10 (55)	79 (575)
Larynx	7 (10)	19 (27)	16 (35)	9 (15)	82 (140)	24 (35)	156 (262)
Lung	43 (58)	86 (110)	54 (97)	41 (60)	473 (699)	109 (144)	807 (1168)
Urinary bladder	15 (39)	41 (98)	23 (112)	14 (44)	180 (603)	56 (146)	328 (1042)
Kidney	2 (13)	9 (45)	4 (50)	3 (21)	66 (537)	12 (70)	95 (736)
Myeloid leukaemia	1 (11)	6 (42)	3 (63)	3 (37)	33 (385)	6 (50)	53 (588)
**Women**							
Lip and oral cavity	1 (4)	2 (21)	0 (15)	0 (4)	10 (145)	0 (4)	13 (193)
Nasopharynx	0 (2)	0 (8)	0 (3)	0 (0)	2 (80)	0 (2)	3 (95)
Pharynx (Hypo+Oro)	0 (0)	0 (0)	0 (4)	0 (0)	6 (40)	0 (3)	6 (47)
Oesophagus	0 (4)	1 (11)	0 (15)	0 (4)	4 (98)	0 (10)	5 (142)
Stomach	0 (14)	1 (30)	0 (56)	0 (12)	2 (119)	0 (54)	3 (285)
Colorectum	0 (48)	0 (157)	0 (106)	0 (42)	2 (1159)	0 (225)	3 (1737)
Liver	0 (10)	1 (32)	0 (27)	0 (5)	3 (225)	0 (33)	5 (332)
Pancreas	1 (12)	1 (35)	0 (21)	0 (7)	3 (150)	1 (78)	6 (303)
Larynx	0 (0)	1 (4)	0 (3)	0 (0)	2 (15)	0 (2)	3 (24)
Lung	7 (23)	11 (48)	1 (15)	1 (13)	38 (223)	4 (45)	62 (367)
Uterine cervix	1 (19)	2 (59)	0 (77)	0 (19)	8 (316)	1 (108)	13 (598)
Urinary bladder	1 (8)	1 (18)	0 (18)	0 (6)	6 (121)	1 (24)	9 (195)
Kidney	0 (5)	0 (17)	0 (17)	0 (8)	2 (228)	0 (32)	3 (307)
Myeloid leukaemia	0 (7)	1 (37)	0 (23)	0 (11)	4 (304)	0 (40)	5 (422)
**Total**	107	286	172	107	1537	328	2536

**All Cancers**	(435)	(1255)	(1424)	(531)	(10133)	(1782)	(15560)

## DISCUSSION

Results of the current analyses indicate that tobacco smoking was responsible for 16.3% (n=2536) of cancer cases in the GCC countries in 2018, with a wide gap between males (22.8%) and females (2.8%). These results are similar to the results reported by Poirier et al.^8^ who determined that 15.7% of cancer cases in Alberta, Canada, were attributable to smoking. The estimates are also comparable to those reported in the UK where 15.1% of cases were attributable to tobacco smoking^10^. Similarly, Cao et al.^9^ estimated that tobacco smoking is responsible for 19% of cancer cases in France. Kristina et al.^16^ determined that 28% of incident cancer cases in South–East Asian (ASEAN) countries (43.3% in males and 8.5% in females) could be attributed to tobacco smoking. However, the higher estimates can be explained by the much higher prevalence of tobacco smoking reported in ASEAN countries (reaching 71.5% among males and 19.9% among females, in some countries)^16^ compared to the GCC countries.

In the current study, tobacco smoking accounted for 22.8% (n=2396) among males and 2.8% (n=140) among females, of cancer cases. Obviously, the sharp contrast between males and females is related to the much higher prevalence of tobacco smoking among males in the GCC countries^7^. Tobacco smoking is more culturally acceptable for men compared to women, which explains the big gap in prevalence between men and women. Women tend to smoke less, and, if they do smoke, they will be reluctant to disclose their smoking behaviour. Although this notion is still valid, the recent cultural changes in the region, especially in the large cities, are associated with more acceptability of tobacco smoking among women. Another phenomenon that may have an effect on the future trend of tobacco-attributable cancer burden is the increasing spread in waterpipe smoking in the region^17^. Waterpipe smoking is gaining popularity, especially among the youth and women^18^. According to the last national survey in Saudi Arabia, waterpipe smoking has doubled among men (from 3.3% to 7.4%) and almost tripled among women (from 0.5% to 1.28%), between 2005 and 2013^18^. If the current trend in tobacco smoking is not halted or reversed, the morbidity and mortality of tobacco related cancers will continue to increase in the future.

GCC countries showed political commitment to tobacco control with all countries having ratified the WHO Framework Convention on Tobacco Control, and all governments have objectives for tobacco control in their national plans^17^. Recently, GCC countries achieved giant steps in the legislation to curb the tobacco epidemic with most countries introducing pictorial warnings and a 100% excise tax on tobacco products, while some countries implemented a plain packaging policy^19^. Nonetheless, more efforts and actions, especially in the area of education of the public and training of healthcare workers^20^, are still required to reverse trends of the smoking epidemic and to overcome challenges. Laws and regulations need to be monitored to ensure firm implementation and prevent interference seeking reversal or attenuation of these regulations. Tobacco companies have a known history of using all types of strategies to undermine tobacco control efforts in the GCC countries, and most likely will continue to do so^21^. The findings of the current study can inform tobacco control efforts in GCC countries by highlighting cancer burden attributable to tobacco smoking and increasing the support of tobacco control among policy makers and public leaders.

### Limitations

The current study has some limitations. First, the estimates are based on the prevalence estimates of tobacco smoking reported for each country. Under-reporting of smoking behaviour, which is expected in household surveys due to social desirability bias, especially among the youth and females, may have led to the underestimation of tobacco-related burden of cancer incidence. Second, estimates of cancer cases attributable to secondhand smoking (household and occupational exposure) could not be calculated because prevalence data of appropriate quality were not available for the GCC countries. If secondhand smoking were included in the current analysis, the estimates of tobacco-attributable cancer incidence would have been higher than the current estimates. Third, the analysis was based on the underlying data retrieved from various sources and would depend on the quality and availability of these data. For example, relative risk information was not available for all types of cancer by sex and no local studies reporting relative risk estimates were available. Also, GLOBOCAN data depend on the reported data from each country registry and would be affected by the completeness of registry data. Finally, a 10-year latency period was used across types of cancer although various latency periods have been reported for different types of cancer. However, a common latency period was used due to the availability of prevalence data and also to follow the approach used in previous studies conducted elsewhere^10,16^.

## CONCLUSIONS

Tobacco smoking accounted for a large portion of cancer cases attributable to preventable risk factors in GCC countries. Preventive efforts focusing on reducing tobacco smoking should be a high priority in GCC countries because they will reduce the incidence of a wide range of cancer types and consequently reduce cancer-related morbidity and mortality.
